# Urinary Microbiota in Female Patients With Dry and Wet Overactive Bladder (OAB)

**DOI:** 10.1155/ijog/5936606

**Published:** 2026-07-20

**Authors:** Xiao Chen, Hui Zou, Jia Wang, Yiran Sun, Mingxia Zhang, Hao Hu, Kexin Xu

**Affiliations:** ^1^ Department of Urology, Shaanxi Provincial People’s Hospital, Xi’an, Shaanxi, China, spph-sx.com; ^2^ Department of Urology, Peking University People′s Hospital, Beijing, China, pku.edu.cn; ^3^ Department of Endocrine, Peking University People’s Hospital, Beijing, China, pku.edu.cn

**Keywords:** 16S rRNA, *Gardnerella*, OAB, urge urinary incontinence, urinary microbiota

## Abstract

**Purpose:**

This study is aimed at comparing the urinary microbiota between OAB patients and healthy controls and at assessing whether urinary incontinence status affects the urinary microbiota in OAB patients.

**Methods:**

From April 2021 to October 2022, female patients admitted to our hospital in Beijing with an Overactive Bladder Symptom Score (OABSS) greater than 3 were selected. Healthy female individuals were chosen as the control group. Urine specimens were collected and subjected to 16S rRNA sequencing. The data obtained were analyzed using bioinformatics techniques.

**Results:**

The average age, OABSS score, and alpha diversity index in the OAB group were significantly higher than those in the control group. At the genus level, the most abundant genus in the OABWet group was Gardnerella (19.69%), and the most abundant genus in the OABDry group was Lactobacillus (12.26%). In the control group, the most abundant genus was also Lactobacillus (19.90%). The OABWet group had significantly higher relative abundances of Gardnerella and Ciliophora compared to the OABDry group, and Ciliophora was more abundant in the control group. Veillonella was less abundant in the OABDry group, while Prevotella and Pseudomonas were less abundant in the control group. Gardnerella was also less abundant in the OABDry group than in the control group.

**Conclusion:**

The urinary microbiota of female OAB patients showed lower species richness and lacked dominant bacterial communities compared to the healthy control group. In wet‐type OAB patients, there was not only a decreased abundance of Lactobacillus but also a high abundance of Gardnerella in the urine.

## 1. Introduction

Overactive bladder (OAB) involves urgency, often with urinary frequency and nocturia, with or without urge urinary incontinence, excluding acute urinary tract infections (UTIs) or other localized bladder and urethral disorders [1, 2]. The etiology of OAB remains unclear. The main pathogenetic hypotheses include the myogenic hypothesis, the neurogenic hypothesis, and the autonomous bladder theory. Epidemiological studies show that over 10% of adults are affected by OAB [3]. Furthermore, the prevalence of OAB increases with age and affects males and females similarly [4]. OAB significantly impacts patients’ quality of life, often requiring strategies like fluid intake control to manage symptoms. While OAB symptoms disrupt daily life, they also cause severe psychological issues [5]. Common risk factors for OAB include lower urinary tract anatomical or neurological abnormalities, obesity, smoking, caffeine consumption [6], and Type 2 diabetes [7, 8], which is also an independent risk factor.

In 2012 [9], the application of new sequencing techniques in urine research disproved the traditional notion that “healthy human urine is sterile.” Further research [10, 11] has shown that urinary microbiota is closely associated with various lower urinary tract diseases and bladder tumors [12]. Additionally, both physical ailments [13] and emotional changes [14, 15] can influence the composition of urinary microbiota populations. Microbial communities in the bladder significantly influence lower urinary tract symptoms [16, 17], but the link between OAB and urinary microbiota needs more research [18]. Research by Moore et al. reported pyuria in 6% of treatment‐naïve OAB patients versus only 1% of controls [19], with this disparity becoming even more pronounced in recurrent OAB patients (39% vs. 1%) [20]. Additionally, recurrent UTI patients exhibited higher rates of detrusor overactivity (DO) compared to controls [21]. In the context of urgency urinary incontinence (UUI) clinical trials, patients showing favorable responses to solifenacin demonstrated lower urinary bacterial abundance and diversity (with a more defined dominant microbiota), while nonresponders harbored multiple bacterial species absent in the responder group [22]. Furthermore, studies on botulinum toxin and anticholinergic therapies indicated that 16S rRNA‐positive OAB patients experienced higher baseline UUI episodes and achieved superior therapeutic outcomes [23].

From April 2021 to October 2022, we studied female patients from our hospital’s Endocrinology Department with OAB and a healthy female control group. We used 16S rRNA sequencing technology to investigate differences in the urinary microbiota between patients with OAB and healthy control subjects, as well as to examine the impact of urinary incontinence on the urinary microbiota in OAB patients.

## 2. Methods

### 2.1. Sample Collection

For this study, female inpatients from our hospital’s Endocrinology Department between April 2021 and October 2022 were selected. Patients with an OABSS score of over 3 were included in the OAB group, while healthy females constituted the control group.

The inclusion criteria for the Experimental Group (OAB) are as follows: (1) age greater than 18 years, (2) OABSS score equal to or greater than 3, and (3) negative results for urine routine and urine culture.

The inclusion criteria for the control group are as follows: (1) age greater than 18 years, (2) clinical exclusion of lower urinary tract disorders, and (3) negative results for urine routine and urine culture.

The exclusion criteria are as follows: (1) age less than 18 years, (2) history of neurogenic bladder or urinary tract malformations, (3) history of malignancy or organ transplantation, (4) undergoing or having a history of radiation or chemotherapy, (5) history of prolonged ureteral stenting or indwelling catheters, (6) pregnancy or lactation status, and (7) exclusion criteria included positive urinalysis results (glucosuria, proteinuria, or UTI with bacterial count > 10^4^ CFU/mL) and the presence of other diseases that may influence the urinary microbiota.

### 2.2. Clinical Data Collection

For the OAB group, we collected OABSS scores, AUASS/IPSS total scores, and quality of life assessments and assessed the presence of urinary incontinence symptoms through medical history inquiry and physical examination. For the control group, only age was recorded.

### 2.3. Urine Specimen Collection

Urine specimens were collected from participants using sterile techniques: Fresh midstream clean‐catch urine (> 25 mL) was collected into sterile cups, preserved in 50 mL centrifuge tubes, and quickly frozen at −80°C. Each participant provided two samples for accuracy.

### 2.4. DNA Extraction and PCR Amplification

Microbial community genomic DNA was extracted from urine samples using the E.Z.N.A. Soil DNA Kit (Omega Bio‐tek, Norcross, Georgia, United States) following the manufacturer’s instructions. DNA quality was assessed with a NanoDrop 2000 UV‐vis spectrophotometer (Thermo Scientific, Wilmington, United States). The V3–V4 hypervariable region of the bacterial 16S rRNA gene was amplified using primers 338F (5 ^′^‐ACTCCTACGGGAGGCAGCAG‐3 ^′^) and 806R (5 ^′^‐GGACTACHVGGGTWTCTAAT‐3 ^′^) on an ABI GeneAmp 9700 PCR thermocycler. PCR conditions were initial denaturation at 95°C for 3 min, 27 cycles of 95°C for 30 s, 55°C for 30 s, 72°C for 45 s, final extension at 72°C for 10 min, and hold at 4°C. The PCR mixture included 4 *μ*L, 2.5 mM dNTPs 2 *μ*L, forward primer (5 *μ*M) 0.8 *μ*L, reverse primer (5 *μ*M) 0.8 *μ*L, TransStart FastPfu DNA Polymerase 0.4 *μ*L, template DNA 10 ng, and finally ddH2O up to 20 *μ*L. PCR was done in triplicate. The products were extracted from a 2% agarose gel, purified with the AxyPrep DNA Gel Extraction Kit (Axygen Biosciences, Union City, California, United States), and quantified using a Quantus Fluorometer (Promega, United States).

### 2.5. Illumina MiSeq Sequencing

Purified amplicons were pooled in equimolar and paired‐end sequenced on an Illumina MiSeq PE300 platform/NovaSeq PE250 platform (Illumina, San Diego, United States) according to the standard protocols by Majorbio Bio‐Pharm Technology Co. Ltd. (Shanghai, China).

### 2.6. Processing of Sequencing Data

The raw 16S rRNA gene sequencing reads were processed as follows: demultiplexed, quality‐filtered using fastp Version 0.20.0 [24], and merged by FLASH Version 1.2.7 [25]. Criteria included the following: (i) 300 bp reads were truncated at sites with an average quality score < 20 over a 50 bp sliding window, and reads shorter than 50 bp or containing ambiguous characters were discarded; (ii) only overlapping sequences longer than 10 bp were assembled with a maximum mismatch ratio of 0.2 in the overlap region; and (iii) samples were distinguished by barcode and primers, with exact barcode matching and up to two nucleotide mismatches in primer matching.

Operational taxonomic units (OTUs) with a 97% similarity cutoff [26, 27] were clustered using UPARSE Version 7.1 [26], and chimeric sequences were identified and removed. The taxonomy of each OTU representative sequence was analyzed by RDP Classifier Version 2.2 [28] against the 16S rRNA database using a confidence threshold of 0.7. All datasets have been uploaded to http://zenodo.org with the DOI10.5281/zenodo.12789785.

### 2.7. Statistical Methods

Data analysis was conducted using SPSS 24.0. The following statistical tests were used: independent sample *t*‐test for unpaired data, paired sample *t*‐test for paired data, one‐way ANOVA for group comparisons, Mann–Whitney *U* test for unpaired data not normally distributed, Kruskal–Wallis *H* test for group comparisons of unpaired data not normally distributed, Wilcoxon signed‐rank test for paired data not normally distributed, and Friedman signed‐rank test for group comparisons of paired data not normally distributed. Post hoc comparisons were conducted using the Nemenyi method when significant differences were found. All *p* values were two‐tailed, with values less than 0.05 considered statistically significant.

### 2.8. Ethical Approval

This study was approved by the Hospital Ethics Committee (Approval No. 2020PHB309), and the approval document is provided in the Supporting Information.

## 3. Results

### 3.1. Specimen Sequencing Results

A total of 27 OAB and 26 control samples were sequenced, yielding 3,276,481 reads. After denoising and removing chimeras, 5620 OTUs were clustered. The 53 samples had a minimum of 17,559 reads, a median of 47,163 reads, an average of 61,820 reads, and a maximum of 241,499 reads.

For the OAB wet group (*n* = 17), the inclusion criteria were an OABSS score > 3 accompanied by urinary incontinence. For the OAB dry group (*n* = 10), the inclusion criteria were an OABSS score > 3 without urinary incontinence.

### 3.2. Basic Clinical Characteristics of Enrolled Participants

A total of 27 OAB samples were sequenced, including 17 OABWet (*n* = 17) and 10 OABDry (*n* = 10) cases. The control group (*n* = 26) had 26 patients.

**Figure 1:**
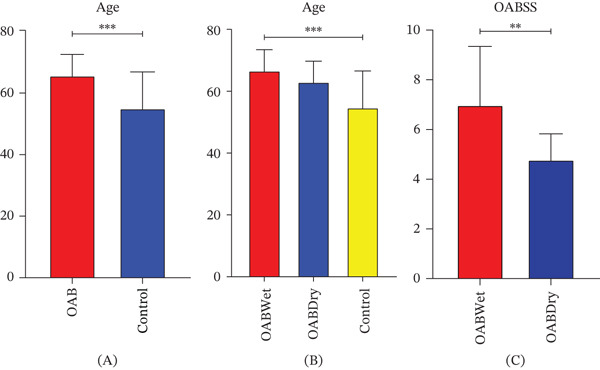
Age differences among different groups. (A) The average age of the OAB group was significantly higher than that of the control group (*p* = 0.0002 < 0.01). (B) The average age of the OABWet group was significantly higher than that of the control group (*p* = 0.0005 < 0.01). (C) The OABWet group had significantly higher OABSS scores compared to the OABDry group (*p* = 0.0098 < 0.01). In the figure, data are presented as mean ± SD. **p < 0.01; ***p < 0.001.

The average ages were 65.8 ± 6.7 years for the OAB group, 67.2 ± 6.6 years for OABWet, 63.5 ± 6.0 years for OABDry, and 55.15 ± 11.78 years for the control group (Figure 1A,B and Figure S1A,B). The OAB group was significantly older than the control group (65.8 vs. 55.15, *p* = 0.0002). Similarly, the OABWet group was older than the control group (67.18 vs. 55.15, *p* = 0.0005). The OABWet group had an average OABSS score of 7.0 ± 2.4, significantly higher than the OABDry group’s 4.8 ± 1.0 (*p* = 0.0098, Figure 1C and Figure S1C). There was no significant difference in QoL scores between OABWet and OABDry. These results indicate that older patients are more likely to experience wet OAB, with more severe symptoms, suggesting the need for more proactive intervention.

### 3.3. Analysis of Urinary Microbiota Species Diversity

**Figure 2:**
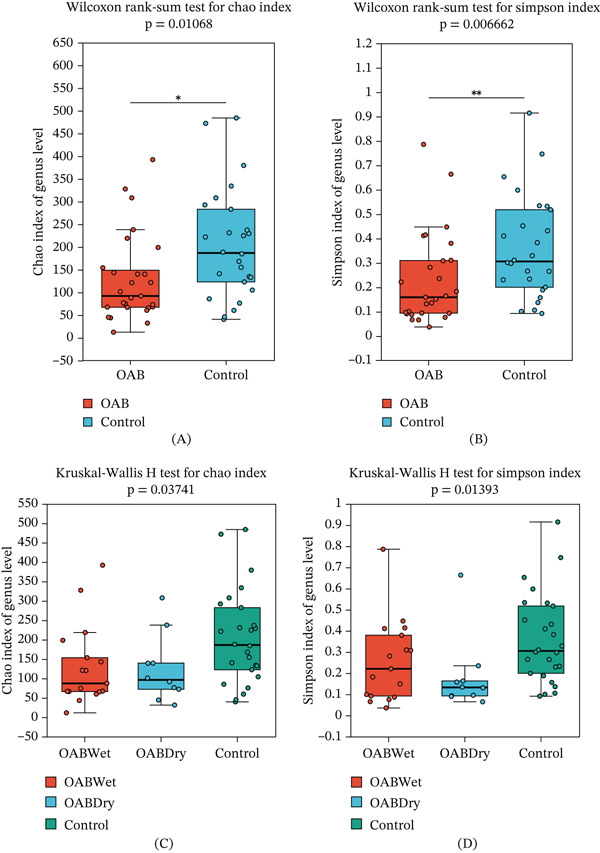
Alpha diversity analysis of urinary microbiota in overactive bladder (OAB) and control groups (diabetes group). (A) Chao index comparison between the OAB group and the control group. (B) Simpson index comparison between the OAB group and the control group. (C) Chao index of the OABWet group, OABDry group, and control group. (D) Simpson index of the OABWet group, OABDry group, and control group.

Alpha diversity is used to describe the number of microbial species in experimental samples and the proportion each species occupies in the overall population. The OAB and control groups showed significant differences in the Chao index (OAB 128.66 vs. control 205.39, Figure 2A and Figure S2A) and Simpson index (OAB 0.22965 vs. control 0.36135, Figure 2B and Figure S2B) (*p* < 0.05), indicating lower alpha diversity in the OAB group.

To further explore the diversity of urinary microbiota in OAB patients, as mentioned earlier, OAB patients were divided into two groups based on the presence of urinary incontinence: OABWet and OABDry. The Chao index was lower in the OABWet group than the control (OABWet 250.6 vs. OABDry 341.4 vs. control 545.9, Figure 2C and Figure S2C), and the Simpson index was lower in the OABDry group than the control (OABWet 0.2123 vs. OABDry 0.1564 vs. control 0.3343, Figure 2D and Figure S2D). This suggests lower species richness in OABWet and lower richness and evenness in OABDry compared to the control group.

### 3.4. Selection of Differentially Abundant Urinary Microbiota Taxa

We analyzed microbiota differences between the OAB and control groups at various taxonomic levels. No significant differences were found at the phylum, class, and order levels.

**Figure 3:**
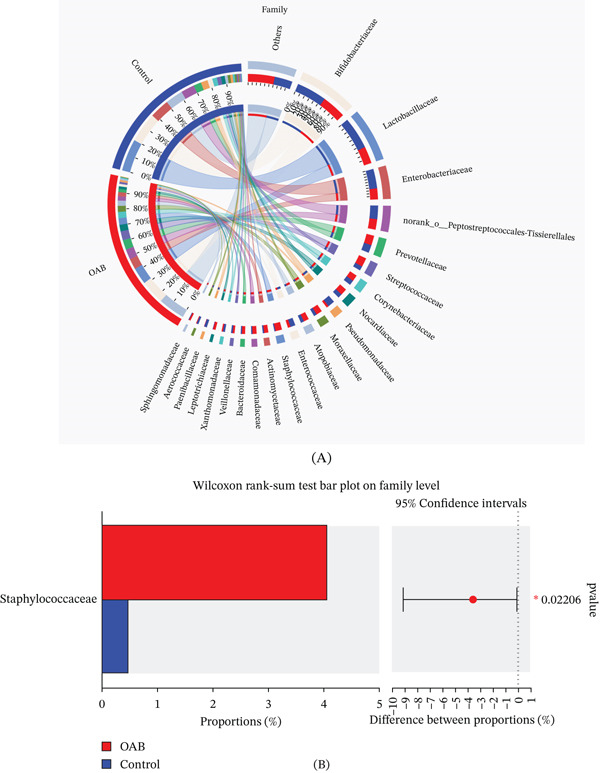
(A) Comparison of urinary microbiota abundance at the family level between the OAB group and the control group. (B) Significantly higher relative abundance of Staphylococcaceae family in the OAB group compared to the control group (5.5% vs. 2.5%). In the figure, data are presented as mean ± SD. **p < 0.01; ***p < 0.001.

In family‐level analysis, the control group had more genera than OAB (496 vs. 400). The highest relative abundance was *Bifidobacteriaceae* in OAB (14.84%) and *Lactobacillaceae* in control (19.91%); *Staphylococcaceae* abundance was higher in OAB (4.06% vs. 0.47%) (Figure S3A–E, Figure 3A,B, and Table S1).

**Figure 4:**
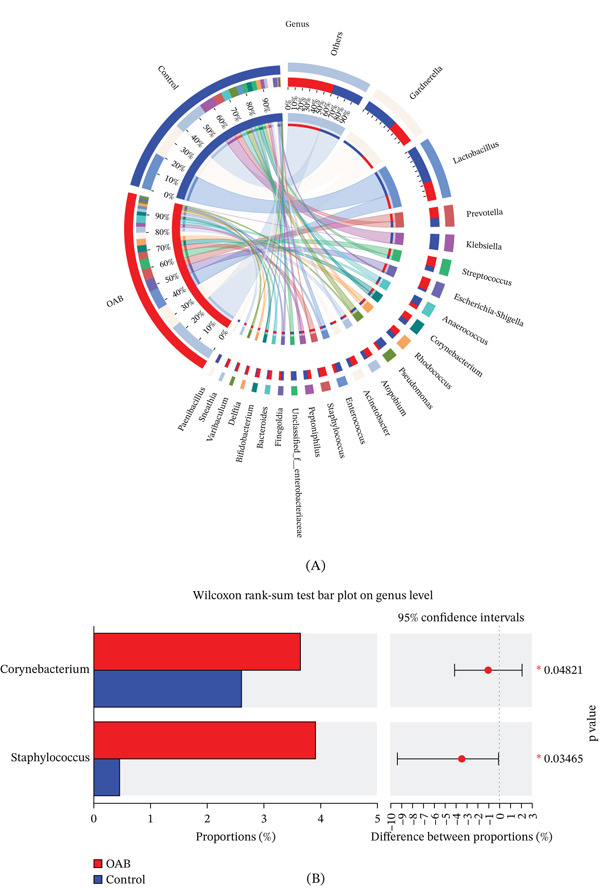
(A) Comparison of urinary microbiota abundance at the genus level between the OAB group and control group. (B) Significantly higher relative abundance of *Staphylococcus* genus (5.4% vs. 2.5%) and *Corynebacterium* genus (3.6% vs. 2.6%) in the OAB group compared to the control group. In the figure, data are presented as mean ± SD. **p < 0.01; ***p < 0.001.

In genus‐level analysis, the control group had more genera than OAB (1052 vs. 838). The highest relative abundance was *Gardnerella* in OAB (12.40%) and *Lactobacillus* in control (19.90%). *Streptococcus* (5.42% vs. 2.52%) and *Corynebacterium* (3.63% vs. 2.61%) were higher in OAB (Figure S3F–H, Figure 4A,B, and Table S2).

Further analysis between OABWet, OABDry, and control groups showed no significant differences at the phylum, class, and order levels.

**Figure 5:**
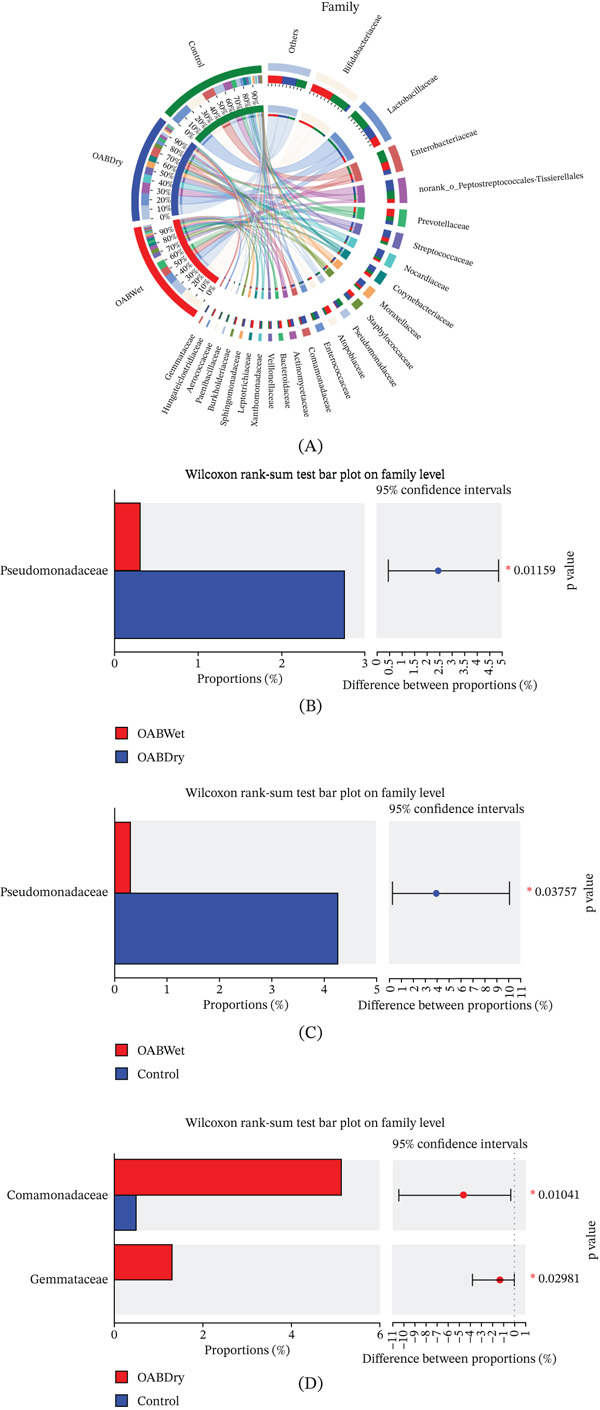
(A) Comparison of relative abundances of urinary microbiota at the family level between OAB and control groups. (B) Significantly higher relative abundance of Pseudomonadaceae family in the dry OAB group compared to the wet OAB group (2.8% vs. 0.31%). (C) Significantly lower relative abundance of Pseudomonadaceae family in the wet OAB group compared to the control group (0.31% vs. 4.3%). (D) Significantly higher relative abundance of Comamonadaceae (5.1% vs. 0.51%) and Gemmataceae (1.3% vs. 0.0011%) families in the dry OAB group compared to the control group. In the figure, data are presented as mean ± SD. **p < 0.01; ***p < 0.001.

The highest family‐level abundance was *Bifidobacteriaceae* in OABWet (22.26%) and *Lactobacillaceae* in OABDry (12.26%) and control (19.91%) (Figure 5A, and Table S3). The higher relative abundances were *Pseudomonadaceae* in OABDry (2.8% vs. 0.31%) and *Comamonadaceae* (5.1% vs. 0.51%) and *Gemmataceae* (1.3% vs. 0.0011%) in OABDry compared to control. *Pseudomonadaceae* was lower in OABWet compared to control (Figure 5B–D).

**Figure 6:**
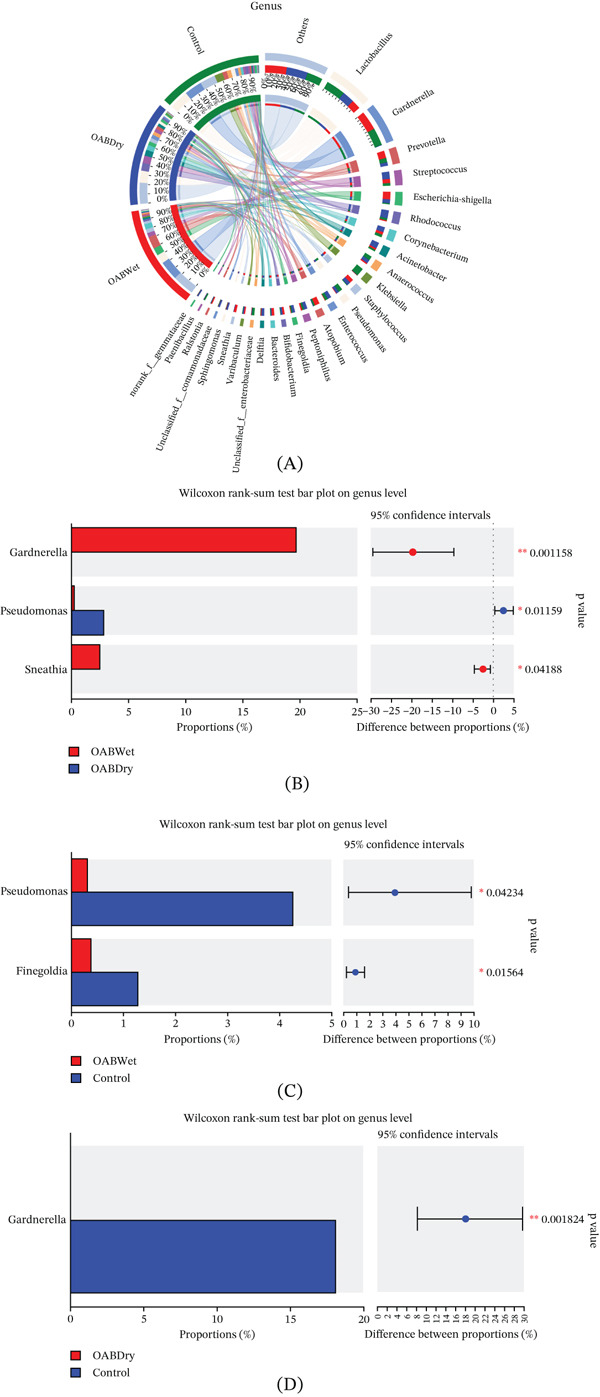
(A) Comparison of relative abundances of urinary microbiota at the genus (genus) level between OAB and control groups. (B) The OABWet group shows significantly higher relative abundances of *Gardnerella* genus (20% vs. 0.0028%) and *Sneathia* genus (2.5% vs. 0%) compared to the OABDry group, while the OABWet group exhibits significantly lower relative abundance of *Pseudomonas* genus (0.31% vs. 2.8%) compared to the OABDry group. (C) The OABWet group displays significantly lower relative abundances of *Pseudomonas* genus (2.8% vs. 4.3%) and *Finegoldia* genus (0.38% vs. 1.3%) compared to the control group. (D) The OABDry group exhibits significantly lower relative abundance of *Gardnerella* genus (0.0011% vs. 18%) compared to the control group. In the figure, data are presented as mean ± SD. **p < 0.01; ***p < 0.001.

The genus‐level analysis was as follows: number of genera: OABWet (682), OABDry (522), and control (1052). The highest relative abundance was *Gardnerella* in OABWet (19.69%) and *Lactobacillus* in OABDry (12.26%) and control (19.90%) (Figure 6A and Table S4). The higher relative abundances were *Gardnerella* (20% vs. 0.0028%) and *Sneathia* (2.5% vs. 0%) in OABWet compared to OABDry. *Pseudomonas* was lower in OABWet compared to OABDry. *Sneathia* (2.5% vs. 0%) was higher in OABWet compared to control. *Finegoldia* was lower in OABWet compared to control. *Norank_f__Gemmataceae* was higher in OABDry compared to control (Figure 6B–D).

## 4. Discussion

Since 2012, new sequencing technologies have uncovered a complex microbial ecosystem, the urinary microbiota, in healthy individuals’ urine [9]. Studies have linked urinary microbiota to various lower urinary tract diseases [10–12]. The composition and excretion of urine significantly impact bladder physiology, highlighting the balance between urinary microbiota and the host as a crucial research focus [29]. A healthy urinary microbiota maintains urine function and quality, while urine composition affects microbiota stability [30]. For example, virulence‐associated genes show significant expression changes when cultured in urine components using MARS [31]. Women’s urinary microbiota, dominated by *Lactobacillus*, *Streptococcus*, and *Gardnerella*, resembles vaginal microbiota [16, 32]. *Lactobacillus*, found in the vaginal environment and on skin, respiratory, intestinal, and urinary tract surfaces, maintains vaginal pH and protects against UTIs [33].

Previous research on the relationship between OAB and urinary microbiota is inconclusive. Pearce et al. and Chen et al. found no significant differences between the urinary microbiota of OAB patients and normal individuals [23, 34]. However, Wu et al. reported higher relative abundances of *Aerococcus*, *Sneathia*, *Proteus*, *Helcococcus*, *Enterococcus*, *Lactococcus*, and *Ureaplasma* in OAB patients, with decreased *Lactobacillus* and lower alpha diversity indices. Additionally, OAB patients’ urinary microbiota showed lower alpha diversity indices (Chao and Simpson) compared to healthy controls [14]. Curtiss et al. found more *Proteus* and fewer *Lactobacillus* in OAB patients, with *Staphylococcus* (59%), *Streptococcus* (51%), *Corynebacterium* (37%), and *Lactobacillus* (28%) being the most common bacteria [22]. Moore et al. reported positive urine cultures in 6% of initially treated OAB patients compared to 1% in controls [19], while Walsh et al. found a higher prevalence in recurrent OAB patients (39% vs. 1%) [20]. These studies suggest a possible, though not direct, link between microbial infection and OAB onset.

This study is aimed at using 16S rRNA technology to investigate the differences in urinary microbiota between OAB patients and healthy individuals, seeking new insights for OAB treatment. The sequencing results indicate that the alpha diversity of OAB patients is lower than that of the control group. In the control group, the combined relative abundance of the Top 4 genera exceeded 50%, while in the OAB group, the Top 5 genera combined only reached 40.46%. This suggests that OAB patients have lower relative abundances of the same bacterial populations compared to the control group (e.g., *Lactobacillus* genus, 19.90% vs. 10.37%), indicating that OAB patients’ urine microbiota is more diverse, lacking typical dominant microbial communities. This conclusion aligns with findings from a clinical study on the efficacy of OAB drugs, where patients lacking dominant microbial communities exhibited more pronounced symptoms and poorer response to treatment [35]. This suggests that OAB symptoms may be related to the disruption of the urinary microbiota balance, loss of *Lactobacillus* as a dominant species, and changes in the bladder microenvironment, characterized by decreased microbial diversity and the disappearance of dominant bacterial groups.

In further studies, the OABWet group showed significantly higher OABSS scores than the OABDry group, indicating that wet OAB, due to the presence of UUI, has a greater impact on the quality of life of patients compared to dry OAB without incontinence. In the OABDry group, the relative abundance of *Lactobacillus* is lower than in the control group but higher than in the OABWet group. In the OABWet group, the relative abundance of *Lactobacillus* is lower than both the OABDry and control groups. *Gardnerella*’s relative abundance is significantly higher in the OABWet group and acts as a dominant microbial group, resembling the urinary microbiota in patients with UUI. In the control group, despite the presence of a high abundance (19.90%) of *Lactobacillus*, there is also a relatively high abundance of *Gardnerella* (18.11%). However, urinary symptoms are not present, suggesting that the protective role of the high abundance of *Lactobacillus* may be significant in the context of urinary incontinence [10].

The female urethral microbiota and vaginal microbiota exhibit a high degree of overlap, predominantly consisting of *Lactobacillus*, *Streptococcus*, and *Gardnerella* species [16, 36]. *Lactobacillus* is one of the dominant commensal bacterial genera in the female vagina and is also present on environmental surfaces and human body surfaces, as well as in the respiratory tract, intestinal tract, urinary tract, and vagina [36, 37]. The normal urethral flora in females is predominantly composed of *Lactobacillus*, *Gardnerella*, and *Streptococcus* species, which largely overlap with the beneficial microbiota of the female vagina [36]. When exogenous bacteria enter the urethra in large numbers and proliferate extensively, UTI occurs. Studies on the pathogenesis of UTI in women have demonstrated that the primary causative bacteria in most cases of female UTI and pyelonephritis originate from the intestinal tract [38]. The infectious process typically begins with intestinal bacteria first contaminating the periurethral area, followed by colonization within the vaginal introitus and around the urethra, ultimately ascending via the urethra to reach the bladder [38, 39].

*Gardnerella* species are commonly isolated pathogens in female bacterial vaginosis [33] and have been identified through 16S rRNA sequencing in urine samples [34]. Typically found in the female urethra, *Gardnerella* is not generally linked to UTIs or lower urinary tract diseases. However, in rare cases, *Gardnerella* infections can lead to severe systemic infections, which are hard to detect with conventional bacterial culturing methods [40, 41]. Studies suggest *Gardnerella* can cause more severe urethral damage than previously thought. Chronic tissue inflammation has been observed in bladder tissue biopsies of OAB patients, with significantly higher levels of cellular inflammatory factors and chemokines in urine compared to the control group. Serum concentrations of inflammatory factors and chemokines, such as interleukin 4, TNF‐*α*, macrophage inflammatory protein‐1*β*, serum amyloid A, and Tie2, were similarly altered compared to healthy controls [42–44].

*Gardnerella* infections are closely linked to bladder function, with increased expression of bladder cholinergic receptors and bradykinin (BK) receptors associated with detrusor muscle overactivity [45, 46]. *Gardnerella* can also directly induce the expression of the nicotinic cholinergic receptor Chrnb4 in bladder afferent neurons, essential for nicotine‐induced bladder contraction in vitro [47]. In mice, brief *Gardnerella* infections can cause the shedding of urinary tract epithelium and increase bladder susceptibility to uropathogenic *Escherichia coli* (UPEC) [48]. Therefore, the incidence of *Gardnerella*‐related UTIs may be significantly underestimated [49].

The urine microbiota of OABWet patients shows a loss of dominant lactic acid bacteria, a higher proportion of *Gardnerella*, and a high abundance of bacteria associated with UTIs, resembling the microbiota in UUI [10, 16, 17]. Similar to OAB treatment, UUI patients with a defined dominant microbial community respond better to drug therapy, while those lacking such a community exhibit poorer treatment outcomes [50].

Based on the above results, it can be inferred that the role of urinary microbiota in the occurrence of OAB may be as follows: *Lactobacillus* plays a protective and stabilizing role in the female lower urinary tract microenvironment, while the urinary microbiota microenvironment in OAB patients is disrupted, leading to localized bladder inflammation, ultimately triggering bladder dysfunction in patients.

*Gardnerella* in the bladder can enhance the susceptibility to UPEC [48]. When UPEC dies and lyses, it produces lipopolysaccharide (LPS), which can activate the TRPA1 channel in sensory neurons, leading to neurogenic inflammation and pain [43, 46]. LPS also interacts with Toll‐like receptor 4 (TLR4) on cells [44] and activates the TRPA1 ion channel [43], inducing inflammation‐related pain independently of TRPA1 receptor activation. Biopsies of tissues from OAB patients reveal chronic, unexplained inflammation, and levels of inflammatory cytokines and chemokines in urine are significantly higher in the OAB group compared to the control group [42]. These findings indicate that inflammation is a critical component of the pathological processes underlying OAB.

This study has limitations mainly due to the inherent constraints of 16S rRNA gene sequencing. This technique focuses solely on bacterial sequencing and cannot explore other microorganisms such as viruses and fungi. Additionally, our investigation into the urinary microbiota is a cross‐sectional observational study, warranting further prospective, interventional research, as well as in vivo and in vitro experiments, to delve deeper into the interaction patterns between urinary microbiota and the patient’s bladder.

## 5. Conclusion

Our study, utilizing 16S rRNA sequencing technology, demonstrated that the urinary microbiota of patients with OAB differs from that of healthy control subjects. Furthermore, urinary incontinence was identified as an independent factor influencing the composition of the urinary microbiota in OAB patients. These findings lay the foundation for future exploration of OAB subtyping and treatment strategies from the perspective of the urinary microbiota.

## Author Contributions

Hui Zou is the coauthor.

## Funding

The study was funded by Peking University People’s Hospital Research and Development Fund (10000).

## Conflicts of Interest

The authors declare no conflicts of interest.

## Supporting Information

Additional supporting information can be found online in the Supporting Information section.

## Supporting information


**Supporting Information 1** Figure S1: Age differences among different groups. (A) The average age of the OAB group was significantly higher than that of the control group (*p* = 0.0002 < 0.01). (B) The average age of the OABWet group was significantly higher than that of the control group (*p* = 0.0005 < 0.01). (C) The OABWet group had significantly higher OABSS scores compared to the OABDry group (*p* = 0.0098 < 0.01). Figure S2: Alpha diversity analysis of urinary microbiota in overactive bladder (OAB) and control groups (diabetes group). (A) Chao index comparison between the OAB group and the control group. (B) Simpson index comparison between the OAB group and the control group. (C) Chao index of the OABWet group, OABDry group, and control group. (D) Simpson index of the OABWet group, OABDry group, and control group. Figure S3: (A) Significantly higher relative abundance of Staphylococcaceae family in OAB group compared to control group (5.5% vs. 2.5%). (B) Significantly higher relative abundance of *Staphylococcus* genus (5.4% vs. 2.5%) and *Corynebacterium* genus (3.6% vs. 2.6%) in the OAB group compared to the control group. (C) Significantly higher relative abundance of Pseudomonadaceae family in the OABDry group compared to the OABWet group (2.8% vs. 0.31%). (D) Significantly lower relative abundance of Pseudomonadaceae family in the OABWet group compared to the control group (0.31% vs. 4.3%). (E) Significantly higher relative abundance of Comamonadaceae (5.1% vs. 0.51%) and Gemmataceae (1.3% vs. 0.0011%) families in the OABDry group compared to the control group. (F) The OABWet group shows significantly higher relative abundances of *Gardnerella* genus (20% vs. 0.0028%) and *Sneathia* genus (2.5% vs. 0%) compared to the OABDry group, while the OABWet group exhibits significantly lower relative abundance of *Pseudomonas* genus (0.31% vs. 2.8%) compared to the OABDry group. (G) The OABWet group displays significantly lower relative abundances of *Pseudomonas* genus (2.8% vs. 4.3%) and *Finegoldia* genus (0.38% vs. 1.3%) compared to the control group. (H) The OABDry group exhibits significantly lower relative abundance of *Gardnerella* genus (0.0011% vs. 18%) compared to the control group.

**Supporting Information 2** Table S1: Family level, the relative abundances of the five most prevalent bacterial taxa (OAB and control). Table S2: Genus level, the relative abundances of the five most prevalent bacterial taxa (OAB and control). Table S3: Family level, the relative abundances of the five most prevalent bacterial taxa (OABWet, OABDry, and control). Table S4: Genus level, the relative abundances of the five most prevalent bacterial taxa (OABWet, OABDry, and control).

## Data Availability

The original data, due to its large volume, have been publicly stored in the database associated with the following link: https://zenodo.org/uploads/12789785.
