# Supplementary material for: Urinary Microbiota in Female Patients With Dry and Wet Overactive Bladder (OAB)
Source: Int J Genomics. 2026 Jul 20;2026:5936606. doi: 10.1155/ijog/5936606 (PMC13383003; doi:10.1155/ijog/5936606)
Supplement: Supplementary file 1 — Supporting Information 1 Figure S1: Age differences among different groups. (A) The average age of the OAB group was significantly higher than that of the control group (p = 0.0002 < 0.01). (B) The average age of the OABWet group was significantly higher than that of the control group (p = 0.0005 < 0.01). (C) The OABWet group had significantly higher OABSS scores compared to the OABDry group (p = 0.0098 < 0.01). Figure S2: Alpha diversity analysis of urinary microbiota in overactive bladder (OAB) and control groups (diabetes group). (A) Chao index comparison between the OAB group and the control group. (B) Simpson index comparison between the OAB group and the control group. (C) Chao index of the OABWet group, OABDry group, and control group. (D) Simpson index of the OABWet group, OABDry group, and control group. Figure S3: (A) Significantly higher relative abundance of Staphylococcaceae family in OAB group compared to control group (5.5% vs. 2.5%). (B) Significantly higher relative abundance of Staphylococcus genus (5.4% vs. 2.5%) and Corynebacterium genus (3.6% vs. 2.6%) in the OAB group compared to the control group. (C) Significantly higher relative abundance of Pseudomonadaceae family in the OABDry group compared to the OABWet group (2.8% vs. 0.31%). (D) Significantly lower relative abundance of Pseudomonadaceae family in the OABWet group compared to the control group (0.31% vs. 4.3%). (E) Significantly higher relative abundance of Comamonadaceae (5.1% vs. 0.51%) and Gemmataceae (1.3% vs. 0.0011%) families in the OABDry group compared to the control group. (F) The OABWet group shows significantly higher relative abundances of Gardnerella genus (20% vs. 0.0028%) and Sneathia genus (2.5% vs. 0%) compared to the OABDry group, while the OABWet group exhibits significantly lower relative abundance of Pseudomonas genus (0.31% vs. 2.8%) compared to the OABDry group. (G) The OABWet group displays significantly lower relative abundances of Pseudomonas genus (2.8% vs. 4. [file IJOG-2026-5936606-s001.docx]

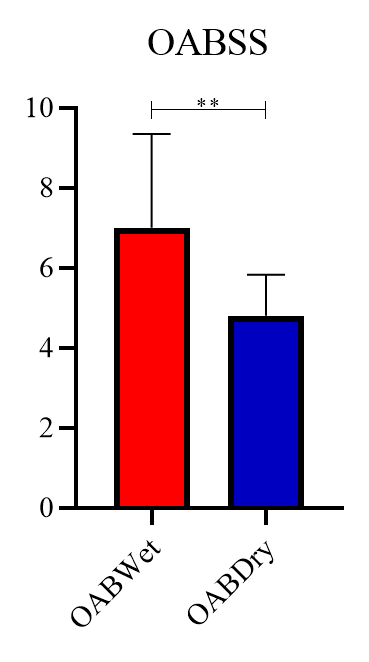

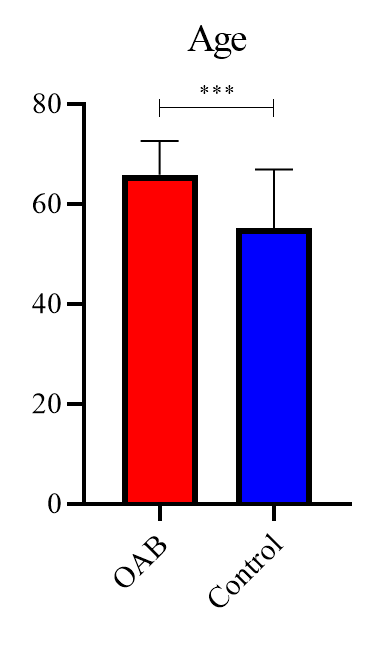

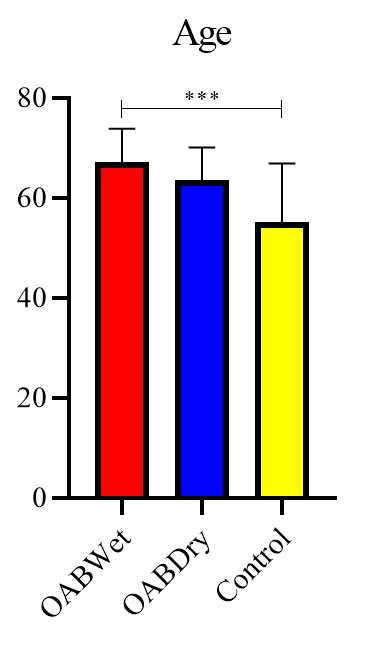

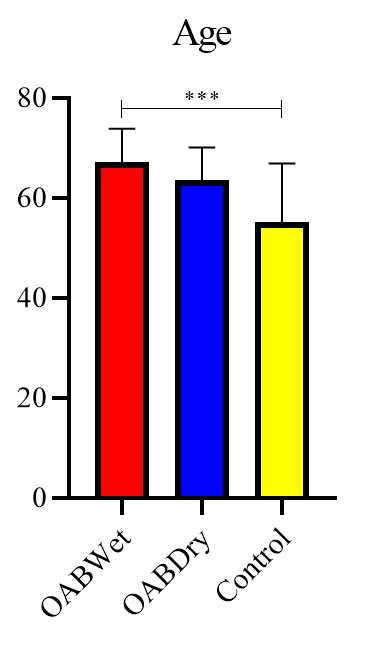


C

B

A

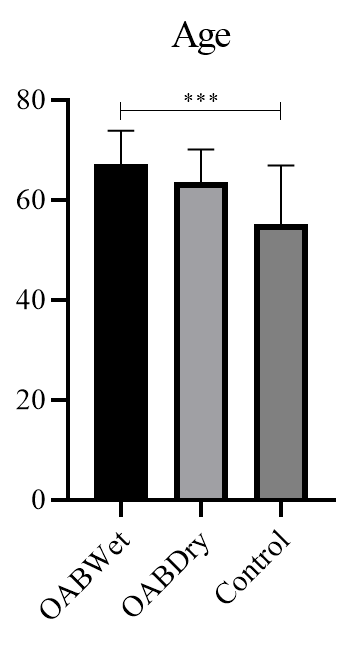


Supplementary material figure 1: Age Differences among Different Groups:

(A): The average age of the OAB group was significantly higher than that of the Control group (P=0.0002<0.01).

(B): The average age of the OABWet group was significantly higher than that of the Control group (P=0.0005<0.01).

(C): The OABWet group had significantly higher OABSS scores compared to the OABDry group (P=0.0098<0.01).


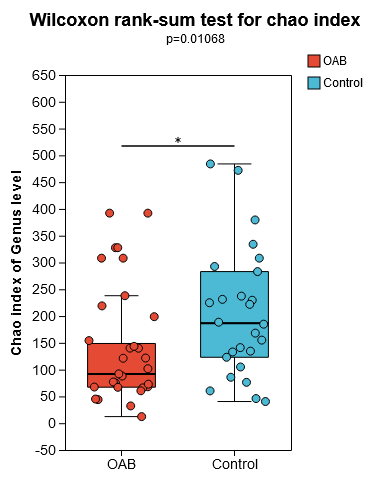

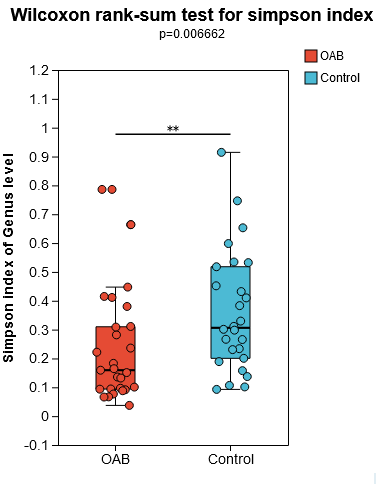


B

A


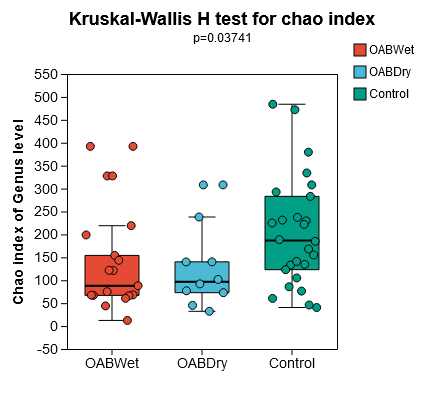

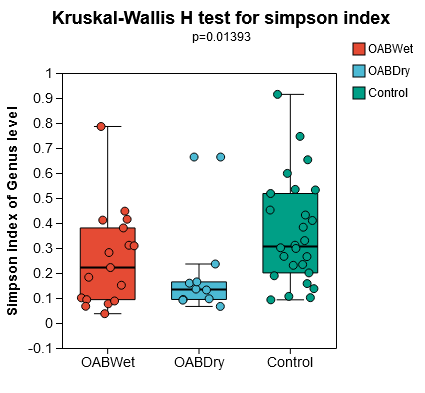


D

C

Supplementary material figure 2: Alpha Diversity Analysis of Urinary Microbiota in Overactive Bladder (OAB) and Control Groups (Diabetes Group)

(A): Chao index comparison between the OAB group and the Control group.

(B): Simpson index comparison between the OAB group and the Control group.

(C) Chao Index of OABWet group, OABDry group, and Control group; (D) Simpson Index of OABWet group, OABDry group, and Control group.


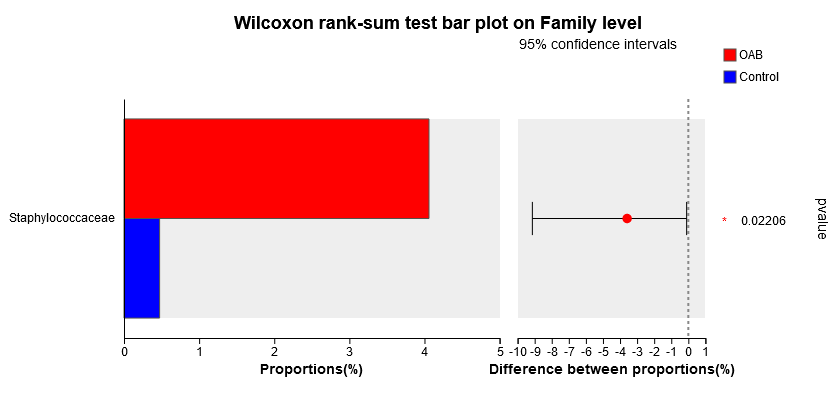


A


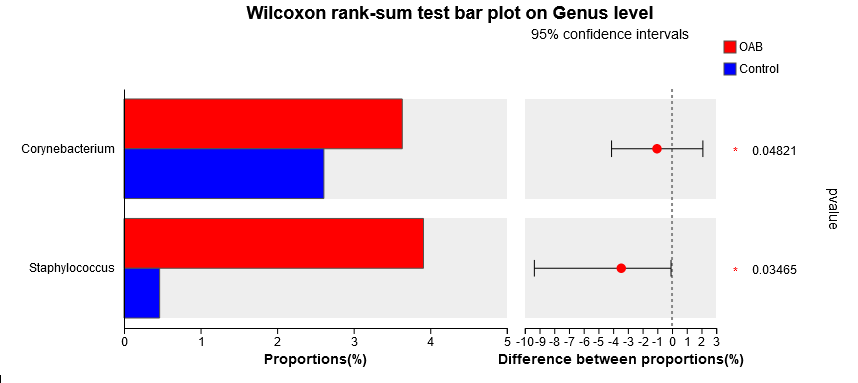


B


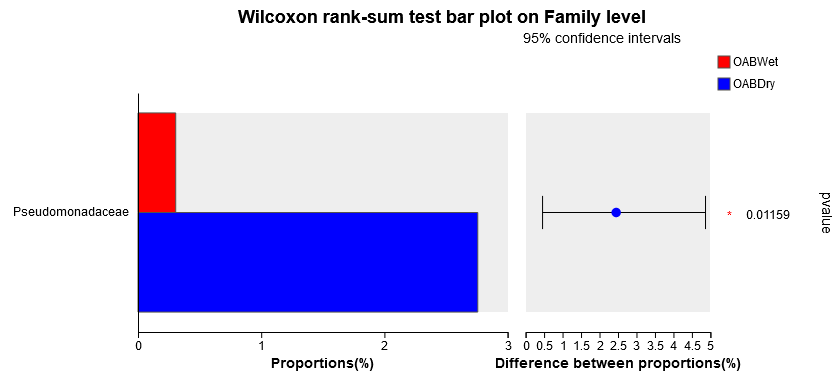


CB


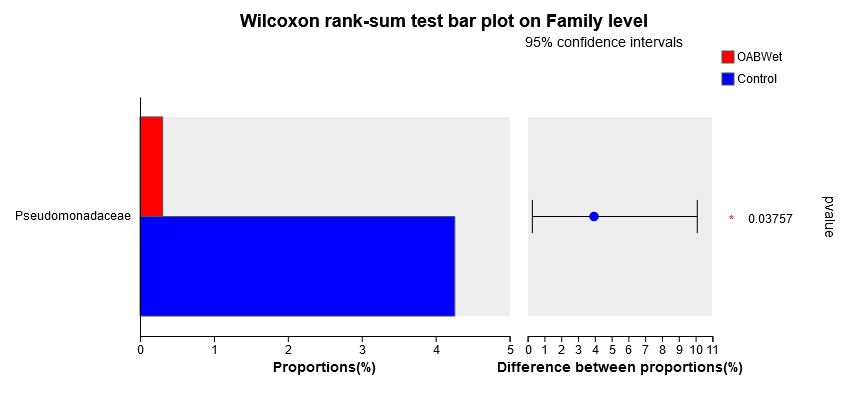


D


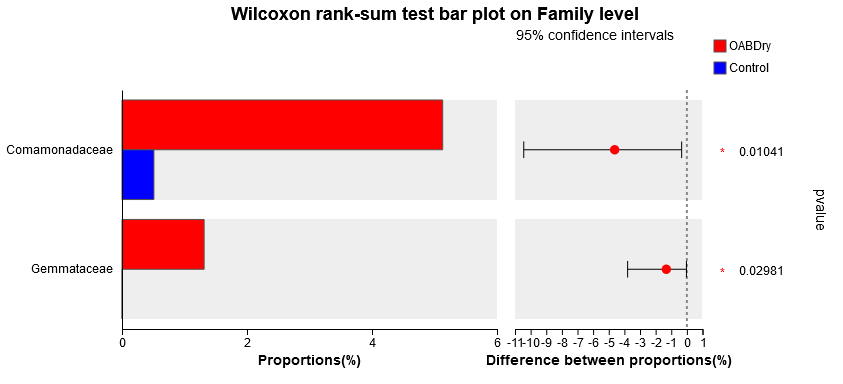


E


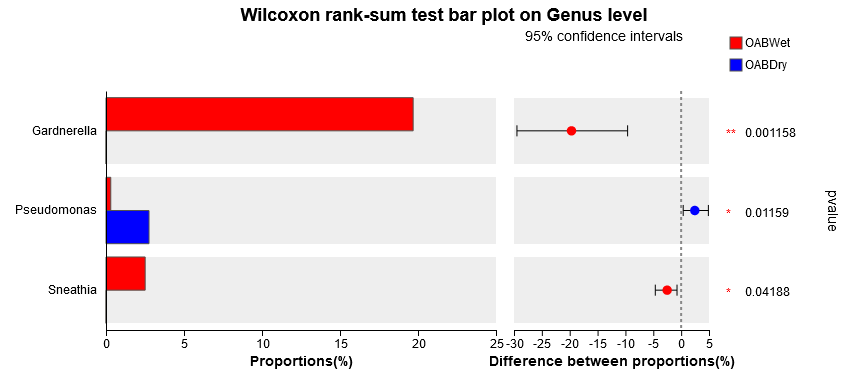


F


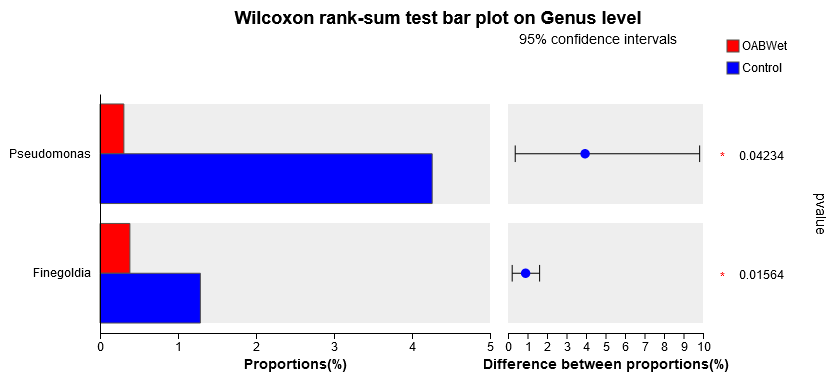


G


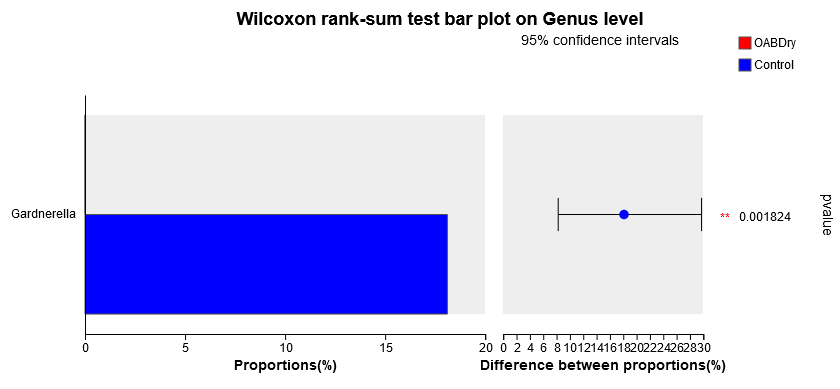


H

Supplementary material figure 3: A) Significantly Higher Relative Abundance of Staphylococcaceae Family in OAB group compared to Control group (5.5% vs 2.5%);B) Significantly Higher Relative Abundance of Staphylococcus Genus (5.4% vs 2.5%) and Corynebacterium Genus (3.6% vs 2.6%) in OAB group compared to Control group; C) Significantly higher relative abundance of Pseudomonadaceae family in the OABDry group compared to the OABWet group (2.8% vs 0.31%); D) Significantly lower relative abundance of Pseudomonadaceae family in the OABWet group compared to the Control group (0.31% vs 4.3%); E) Significantly higher relative abundance of Comamonadaceae (5.1% vs 0.51%) and Gemmataceae (1.3% vs 0.0011%) families in the OABDry group compared to the Control group;F) OABWet group shows significantly higher relative abundances of Gardnerella genus (20% vs 0.0028%) and Sneathia genus (2.5% vs 0%) compared to the OABDry group, while OABWet group exhibits significantly lower relative abundance of Pseudomonas genus (0.31% vs 2.8%) compared to the OABDry group; G) OABWet group displays significantly lower relative abundances of Pseudomonas genus (2.8% vs 4.3%) and Finegoldia genus (0.38% vs 1.3%) compared to the Control group; H) OABDry group exhibits significantly lower relative abundance of Gardnerella genus (0.0011% vs 18%) compared to the Control group.
