# Supplementary material for: Urinary Microbiota in Female Patients With Dry and Wet Overactive Bladder (OAB)
Source: Int J Genomics. 2026 Jul 20;2026:5936606. doi: 10.1155/ijog/5936606 (PMC13383003; doi:10.1155/ijog/5936606)
Supplement: Supplementary file 2 — Supporting Information 2 Table S1: Family level, the relative abundances of the five most prevalent bacterial taxa (OAB and control). Table S2: Genus level, the relative abundances of the five most prevalent bacterial taxa (OAB and control). Table S3: Family level, the relative abundances of the five most prevalent bacterial taxa (OABWet, OABDry, and control). Table S4: Genus level, the relative abundances of the five most prevalent bacterial taxa (OABWet, OABDry, and control). [file IJOG-2026-5936606-s002.docx]

Supplementary material Table1:family level, the relative abundances of the five most prevalent bacterial taxa(OAB,Control)

| **Family** | **OAB (n = 27)** | **Control (n = 26)** |
| --- | --- | --- |
| *Bifidobacteriaceae* | 12.2653541% | 18.6638892% |
| *Lactobacillaceae* | 10.7593726% | 19.8357625% |
| *norank_o__Peptostreptococcales-Tissierellales* | 7.3126568% | 8.3016818% |
| *Enterobacteriaceae* | 6.1271244% | 12.1011798% |
| *Streptococcaceae* | 5.8511472% | 2.5259893% |

Supplementary material Table2:genus level, the relative abundances of the five most prevalent bacterial taxa(OAB,Control)

| **Genus** | **OAB (n = 27)** | **Control (n = 26)** |
| --- | --- | --- |
| *Lactobacillus* | 10.7593726% | 19.8342292% |
| *Gardnerella* | 9.8291139% | 18.164255% |
| *Escherichia-Shigella* | 5.8452009% | 0.9416604% |
| *Streptococcus* | 5.7689202% | 2.5211704% |
| *Prevotella* | 5.4590909% | 4.5705249% |

Supplementary material Table 3:family level, the relative abundances of the five most prevalent bacterial taxa(OABWet,OABDry,Control)

| **Genus** | **OABWet (n = 17)** | **OABDry (n = 10)** | **Control (n = 26)** |
| --- | --- | --- | --- |
| *Bifidobacteriaceae* | 22.2486876% | 2.2820206% | 18.6638892% |
| *others* | 14.4286657% | 14.3732559% | 10.3151134% |
| *Lactobacillaceae* | 9.2498233% | 12.2689219% | 19.8357625% |
| *Enterobacteriaceae* | 7.9664191% | 4.2878296% | 12.1011798% |
| *Prevotellaceae* | 7.7486658% | 3.2040549% | 4.7501391% |

Supplementary material Table 4:genus level, the relative abundances of the five most prevalent bacterial taxa(OABWet,OABDry,Control)

| **Genus** | **OABWet (n = 17)** | **OABDry (n = 10)** | **Control (n = 26)** |
| --- | --- | --- | --- |
| others | 22.2158571% | 21.7580728% | 15.8102135% |
| Gardnerella | 19.6570889% | 1.14E-05 | 18.164255% |
| Lactobacillus | 9.2498233% | 12.2689219% | 19.8342292% |
| Escherichia-Shigella | 7.7687661% | 3.9216356% | 0.9416604% |
| Prevotella | 7.7158354% | 3.2023464% | 4.5705249% |
